# Chimeric Hemagglutinin-Based Live-Attenuated Vaccines Confer Durable Protective Immunity against Influenza A Viruses in a Preclinical Ferret Model

**DOI:** 10.3390/vaccines9010040

**Published:** 2021-01-11

**Authors:** Wen-Chun Liu, Raffael Nachbagauer, Daniel Stadlbauer, Shirin Strohmeier, Alicia Solórzano, Francesco Berlanda-Scorza, Bruce L. Innis, Adolfo García-Sastre, Peter Palese, Florian Krammer, Randy A. Albrecht

**Affiliations:** 1 Department of Microbiology, Icahn School of Medicine at Mount Sinai, New York, NY 10029, USA; wenchun0617@gmail.com (W.-C.L.); raffael.nachbagauer@gmail.com (R.N.); daniel.stadlbauer@mssm.edu (D.S.); shirin.strohmeier@mssm.edu (S.S.); alicia.solorzano.2010@gmail.com (A.S.); Adolfo.Garcia-Sastre@mssm.edu (A.G.-S.); peter.palese@mssm.edu (P.P.); florian.krammer@mssm.edu (F.K.); 2 Global Health and Emerging Pathogens Institute, Icahn School of Medicine at Mount Sinai, New York, NY 10029, USA; 3 Biomedical Translation Research Center, Academia Sinica, Taipei 11571, Taiwan; binnis@path.org; 4 Moderna Therapeutics, Inc., Cambridge, MA 02141, USA; 5 PATH US, Seattle, WA 98121, USA; francesco.x.berlandascorza@gsk.com; 6 GSK Vaccine Institute for Global Health (GVGH), 53100 Siena, Italy; 7 Department of Medicine, Icahn School of Medicine at Mount Sinai, New York, NY 10029, USA; 8 The Tisch Cancer Institute, Icahn School of Medicine at Mount Sinai, New York, NY 10029, USA

**Keywords:** universal influenza virus vaccine, ferret, hemagglutinin stalk antibody, chimeric hemagglutinin, long-lived immunity

## Abstract

Epidemic or pandemic influenza can annually cause significant morbidity and mortality in humans. We developed novel chimeric hemagglutinin (cHA)-based universal influenza virus vaccines, which contain a conserved HA stalk domain from a 2009 pandemic H1N1 (pH1N1) strain combined with globular head domains from avian influenza A viruses. Our previous reports demonstrated that prime-boost sequential immunizations induced robust antibody responses directed toward the conserved HA stalk domain in ferrets. Herein, we further followed vaccinated animals for one year to compare the efficacy and durability of these vaccines in the preclinical ferret model of influenza. Although all cHA-based immunization regimens induced durable HA stalk-specific and heterosubtypic antibody responses in ferrets, sequential immunization with live-attenuated influenza virus vaccines (LAIV-LAIV) conferred the best protection against upper respiratory tract infection by a pH1N1 influenza A virus. The findings from this study suggest that our sequential immunization strategy for a cHA-based universal influenza virus vaccine provides durable protective humoral and cellular immunity against influenza virus infection.

## 1. Introduction

The Advisory Committee on Immunization Practices (ACIP) recommends seasonal influenza virus vaccination for everyone 6 months of age or older [1]. Despite the availability of seasonal influenza virus vaccines, types A and B influenza viruses continue to co-circulate globally in humans [2]. The Centers for Disease Control and Prevention estimated 531,000 to 647,000 hospitalizations and 36,400 to 61,200 deaths in the United States for the 2018–2019 influenza season (30 September 2018–18 May 2019), which was categorized as being of moderate severity [3]. Global seasonal influenza-associated respiratory deaths are estimated to range from 291,000 to 646,000 on an annual basis [4]. Current licensed seasonal influenza virus vaccines are formulated as inactivated influenza virus vaccines (IIV), live-attenuated influenza virus vaccines (LAIV), or recombinant hemagglutinin (HA) proteins [5]. In general, seasonal influenza vaccines provide immunity against infection by influenza viruses that antigenically match the vaccine strains [6,7,8,9,10,11,12,13]. The effectiveness of seasonal influenza vaccines to prevent laboratory-confirmed influenza varies by influenza season (with estimates ranging from 10 to 60%), with an estimate of 29% for the overall effectiveness of the 2018–2019 seasonal influenza vaccines [14]. Antigenic shift or drift, two processes that change the antigenicity of the HA and neuraminidase (NA) surface glycoproteins, can result in an antigenic mismatch between the vaccine and circulating influenza virus strains. This antigenic mismatch can contribute to the reduced effectiveness of seasonal influenza vaccines or potential lack of vaccine efficacy from new viruses emerging from the animal reservoir. In the case of the 2018–2019 influenza season, vaccine effectiveness (VE) was 5% against antigenically drifted clade 3C.3a H3N2 influenza A viruses [14]. As a consequence of antigenic change and variable VE of seasonal influenza vaccines [15], influenza vaccines must be reformulated and re-administered annually based on the influenza virus vaccine strains selected by the World Health Organization [16]. Current seasonal influenza virus vaccines induce protection against infection by antigenically matched influenza viruses; however, IIV vaccines fail to induce robust immunity in the elderly. On the other hand, LAIV, which is only recommended for individuals of 6 months to 49 years of age, can induce more robust and long-lived cellular immunity than IIV. These caveats leave the elderly populations poorly protected [17]. Moreover, seasonal influenza vaccines provide suboptimal protection against antigenically novel pandemic influenza viruses. In response to an outbreak of pandemic influenza, approximately 5–6 months will elapse from initial vaccine strain selection to final distribution of an antigenically matched pandemic vaccine [18,19]. This interval in vaccination coverage leaves the human population, especially high-risk individuals, vulnerable to influenza. 

The U.S. National Institute of Allergy and Infectious Diseases released a directive to develop a universal influenza virus vaccine [20], which should have improved effectiveness in protecting against symptomatic influenza virus infection, protect against cross-subtypes/groups seasonal and pandemic influenza viruses, and confer durable protective immunity that lasts at least one year [21,22,23]. Subbarao and colleagues developed a pandemic H5N1 LAIV based on the Ann Arbor cold-adapted backbone. Immunization of ferrets with this H5N1 LAIV induced long-term protective immunity; however, the breadth of immunity was limited to specific clades of the H5 subtype [24]. Zhan et al. reported that immunization with one or two doses of vaccine induced moderate levels of antibody titers that lasted for approximately 11 weeks, whereas infection induced long-lived immunity that persisted for 30 weeks post-infection [25]. To address the issue of duration of protective immunity, there are several approaches that could be applied to the development of a universal influenza virus vaccine [26,27]. We developed a chimeric hemagglutinin (cHA)-based universal influenza virus vaccine that is designed to focus humoral immune responses on the highly conserved HA stalk domain [28,29]. We previously demonstrated in the ferret model of influenza that our cHA-based approach to a universal influenza vaccine induced HA stalk-specific humoral immunity that conferred protection against infection by human or avian influenza A viruses [30,31,32,33,34]. In agreement with our results, Rudenko and colleagues recently reported that the sequential immunization of ferrets with cHA-based LAIV vaccines induced protective HA stalk-specific antibody responses [35]. However, these ferret studies did not examine the duration of the protective HA stalk-specific humoral immunity, which is one of the critical characteristics of a universal influenza virus vaccine [20].

Genetically outbred ferrets are the most appropriate small animal model of influenza [36], and they were previously used in preclinical studies aimed at examining the immunogenicity and protective efficacy of our cHA-based vaccine [30,31,32,33,34]. Herein, we followed vaccinated ferrets for one year to compare the efficacy and the duration of protective immunity conferred by our cHA-based LAIV-LAIV immunization regimens with that of a LAIV-IIV immunization regimen. Our results demonstrated that sequential cHA-based LAIV-LAIV and LAIV-IIV immunization regimens induced long-lived stalk-specific and broadly reactive humoral and cellular immune responses. As compared to other vaccination regimens examined, the LAIV-LAIV immunization regimen provided the best protection against challenge infection by a 2009 pandemic H1N1 (pH1N1) influenza A virus in ferrets. 

## 2. Materials and Methods 

### 2.1. Ethics Statement

All animal experiments in this study were performed in accordance with an animal protocol (IACUC-2013-1408) approved by the Institution Animal Care and Use Committee (IACUC) of the Icahn School of Medicine at Mount Sinai (ISMMS). All ferrets were housed in a temperature- and humidity-controlled Animal Biosafety Level 2 (ABSL-2) facility. All procedures throughout the study were designed to minimize animal suffering.

### 2.2. Cells, Viruses, and Proteins 

The methods for Madin-Darby Canine Kidney (MDCK) cell culture and plaque assays were described previously [31]. Virus stocks were prepared in 8-day-old specific pathogen-free embryonated chicken eggs (Charles River Laboratories), as previously described [31]. Influenza virus plaques were visualized by immunostaining or crystal violet staining [31]. The cH6/1 (containing H6 head domain from A/mallard/Sweden/81/02 combined with an H1 stalk domain of A/California/04/09 (Cal/09)), H1 (Cal/09), H2 (A/mallard/Netherlands/5/99), H6 (A/mallard/Sweden/81/02), and H18 (A/bat/Peru/33/10) recombinant proteins were produced by a baculovirus expression system as described previously [37,38].

### 2.3. cHA-Based Universal Vaccine and QIV Vaccine Preparations

GlaxoSmithKline (GSK) provided the IIV split virus vaccines, which were described previously [31,33]. All IIV vaccines used in this preclinical study were adjuvanted with Adjuvant System 03 (AS03) as described in our previous studies [31,33,39]. The cH8/1 and cH11/1 LAIVs were reported previously [31,33]. The recombinant influenza B virus expressing cH9/1 virus (B-cH9/1) was as previously described [40]. The quadrivalent inactivated influenza vaccine (QIV), Fluarix, was produced by GSK and it contained the 2016/17 formulation with the strains A/Christchurch/16/2010 (H1N1), A/Hong Kong/4801/2014 (H3N2), B/Brisbane/60/2008, and B/Phuket/3073/2013.

### 2.4. Ferret Immunization and Challenge

**Figure 1:**
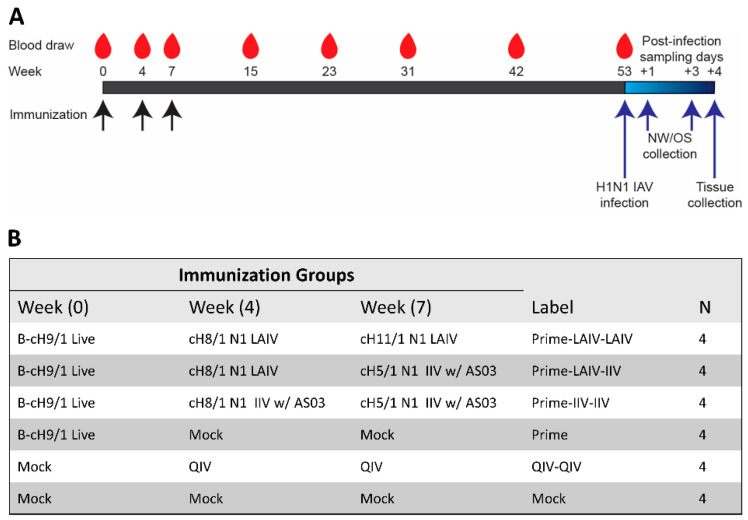
Overview of the longitudinal vaccination study and experimental groups. (**A**) Sequential immunization strategy to induce long-lived H1 stalk immunity. Immunizations (black arrows) and blood collection days (red drops) are indicated. All ferrets were challenge infected with pH1N1 (Cal/09) influenza A virus 379 days post-prime immunization. Nasal wash (NW) and Oropharyngeal swab (OS) specimens were collected on days 1 and 3 post-challenge infection. Blood and tissue specimens were collected on day 4 post-challenge infection. (**B**) Experimental and control groups. All ferrets that received the cHA-based vaccines were primed with the influenza B virus expressing cH9/1 (B-cH9/1) live virus. The primed ferrets next received a booster immunization of cH8/1 N1 LAIV vaccine or AS03-adjuvanted cH8/1 N1 IIV vaccine on day 28 (week 4) post-prime immunization, followed by a second booster immunization with cH11/1 N1 LAIV or AS03-adjuvanted cH5/1 N1 IIV vaccine on day 49 (week 7) post-prime immunization. The “Prime” ferrets received the B-cH9/1 prime immunization followed by booster immunizations with naive allantoic fluid (NAF). The “QIV-QIV” ferrets received mock immunizations with NAF. One group of ferrets that were sequentially immunized with quadrivalent inactivated influenza vaccine (QIV) served as a “standard of care” control. *N* means number of animals for each vaccination group.

Outbred 4-month-old castrated male Fitch ferrets seronegative for prior influenza virus exposure were purchased from Marshall BioResources (North Rose, NY, USA). All ferrets in this study were randomly assigned to the vaccination or control groups. As indicated in the figure legends, each experimental group consisted of four ferrets (*n* = 4). Figure 1 summarizes the sequential immunizations strategies performed in this preclinical study. To establish pre-existing group 1 H1 HA stalk-specific immunity, naive ferrets were prime immunized by intranasal administration of 1 × 10^7^ plaque forming units (PFU) of B-cH9/1 virus as described in our previous study [31]. The intervals of prime-boost vaccinations and detailed immunization regimens are indicated in Figure 1A. The dosage of either cH8/1 or cH11/1 LAIV used in this study was 1 × 10^7^ PFU per animal. Some groups of ferrets were intramuscularly boosted with 0.5mL dose of AS03-adjuvanted cH5/1 IIV. Blood samples were collected at the indicated time points for preparation of sera. A “standard of care” group of ferrets received two human doses of QIV (QIV-QIV, Fluarix, GSK). All ferrets were challenge infected with 10^6^ PFU of Cal/09 pH1N1 virus at 379 days post-prime immunization. For evaluation of vaccine effectiveness, nasal wash and oropharyngeal swab samples were collected from anesthetized ferrets on 1 and 3 days post-challenge infection. On day 4 post-challenge infection, anesthetized ferrets were euthanized by exsanguination followed by intracardiac injection of Sleepaway euthanasia solution (Fort Dodge, Sodium Pentobarbital). Tissue specimens (olfactory bulbs, nasal turbinates, trachea, and lung) were collected from each individual ferret to quantify viral titers by plaque assays.

### 2.5. Enzyme-Linked Immunosorbent Assay (ELISA)

HA-specific and N1 NA-specific antibodies in serum were measured by ELISA with the above-mentioned recombinant proteins. The ELISA was conducted as described in our previous study [31]. In brief, 0.1 μg of antigen was added to each well of 96-well plate, which was then incubated overnight at 4 °C. Plates were washed three times with phosphate buffered saline (PBS) containing 0.1% Tween 20 (PBST) and then blocked with 0.22 mL of blocking solution (PBST containing 3% goat serum (Gibco, New York, NY, USA) and 0.5% milk powder) for 1 h at room temperature (RT). Two-fold diluted serum samples were added onto plates and incubated for 2 h at RT. The plates were then washed three times with PBST, and horseradish peroxidase (HRP)-labeled anti-ferret immunoglobulin G (IgG) was added to each well and incubated for 1 h at RT. After 4× PBST washes, the plates were developed by the addition of the substrate SigmaFast OPD (o-phenylenediamine dihydrochloride) and then the plates were read at a wavelength of 490 nm in a plate reader (Biotek Synergy H1). The mean + 3 × standard deviations (SD) of blank wells were calculated as a cut-off endpoint value. 

### 2.6. Enzyme-Linked Lectin Assay (ELLA) 

The inhibition of N1 NA enzymatic activity in serum was measured by ELLA, which was performed as described in our previous study [31]. We first measured the NA activity of a PR8 × H7N1 virus (H7 HA from duck/mallard/Alberta/24/2001 (H7N1), N1 NA from Cal/09 strain, and the remaining genes from A/PR/8/1934), and determined the optimal concentration of virus used for neuraminidase inhibition (NI) assay. Briefly, 7.5 µg of fetuin (Sigma) was added to each well of 96-well plate, which was then incubated overnight at 4 °C. The next day, the plates were washed once by PBST and blocked with 200 μL of blocking buffer (PBS containing 1% BSA) for 1 h at RT. One hundred (100) µL of two-fold serially diluted virus (starting dilution of 1:5) was then added to the fetuin-coated plates, which were then incubated for 2 h at 37 °C. Next, the plates were washed three times with PBST, and 0.5 µg of HRP-conjugated peanut agglutinin (PNA, Sigma, New York, NY, USA) was added to the plates, which were then incubated for 2 h at RT. The supernatants were aspirated, and the plates washed three times by PBST. Reactions were developed by adding 3, 3’, 5, 5’ tetramethyl benzidine (TMB) substrate. After 15 min, reactions were stopped by the addition of 3M phosphoric acid, and then the plates were read at a wavelength of 410 nm in a plate reader (Biotek). Forty thousand (4 × 10^4^) PFU of influenza virus (approximately half the maximal optical density (OD) reading value) was used for subsequent NI assays. For NI assays, ELISA plates were coated and blocked in the same manner used for the NA assay. While plates were blocking, 125 µL of two-fold serially diluted ferret serum samples (week 15 and pre-challenge time points, starting at a 1:50 dilution) was mixed with 125 µL of virus stock (at a concentration of 4 × 10^4^ PFU) and incubated at 37 °C for 1 h. After blocking, the virus-serum mixtures were then transferred to corresponding fetuin-coated plates. The following steps were identical as for the NA assay described above. The plates were scanned with a Synergy H1 hybrid reader (BioTek, Winooski, VT, USA) to quantify the colorimetric signals, which were then analyzed with Prism 8.0 software as previously described [31]. The ELLA NI titer was defined as the reciprocal serum dilutions inhibiting 50% of viral NA enzymatic activity.

### 2.7. Hemagglutination Inhibition (HI) Assays

The HI assays were performed as described elsewhere [41]. In brief, sera were treated with receptor-destroying enzyme (RDE) (Denka Seiken) for 16–18 h at 37 °C, followed by treatment with 2.5% sodium citrate for 30 min at 56 °C to block non-specific sialic acid binding. PBS was then added to each sample, which resulted in an assay starting dilution of 1:10. Stocks for each influenza virus strain were diluted to a final HA titer of 8 HA units/50 μL in FTA Hemagglutination Buffer (Phosphate Buffered Saline, pH 7.2; BD BBL). Equal volumes of pre-diluted RDE-treated sera were then mixed with working stock of each influenza virus strain and incubated for 30 min at RT to allow any HA-specific antibodies present in the serum to inhibit hemagglutination activity. Next, a 0.5% suspension of turkey red blood cells was added and incubated for 45 min at 4 °C. The HI titer was defined as the reciprocal of the highest dilution of serum that inhibited red blood cell hemagglutination by influenza virus.

### 2.8. Preparation of Single-Cell Suspensions from Ferret Tissues and Peripheral Blood Mononuclear Cells (PBMCs) 

Cell preparations were performed as described in our previous study [31]. Mediastinal lymph nodes (MLN) were excised, dissociated with digestion media (eRPMI media containing DNase I and Type IV collagenase) for 30 min at 37 °C. Next, the enzyme digested-lymph nodes were mechanically rinsed and passed through 70 µm nylon cell strainers (BD falcon, Jersey, NJ, USA). Contaminating red blood cells (RBC) present in the single-cell suspensions were lysed with RBC lysis buffer (Affymetrix Ebioscience, New York, NY, USA). Purified single-cell suspensions of PBMCs and MLN leukocytes were immediately cryopreserved following isolation from tissues or blood for flow cytometric analyses.

### 2.9. Peptide Stimulation and Flow Cytometry Analyses

We immunophenotyped the T cell responses following the procedure described in our previous report [31]. Briefly, single-cell suspensions of PBMCs or MLN leukocytes were thawed and resuspended in complete RPMI media (cRPMI, RPMI 1640, 1 × P/S, 10% heat-inactivated, certified FBS, 10 mM HEPES, 1 mM sodium pyruvate, 1× non-essential amino acid, and 50 µM β-mercaptoethanol). Brefeldin A was also included at 10 µg/mL to inhibit protein secretion. Cells were stimulated overnight with 0.1 µg of pooled PepTivator Influenza A (H1N1) nucleoprotein (Miltenyi Biotec, Bergisch Gladbach, Germany) peptides of Cal/09 (H1N1) or 0.1 µg for each HA stalk region of the Cal/09 (H1N1), respectively, as our previous study described [31]. Data were acquired with a 10 color, 3 laser Gallios Flow Cytometer (Beckman Coulter, Jersey, NJ, USA) with Kaluza Analysis software. All data files were analyzed using FCS Express software, version 7.01 (De Novo Software, Pasadena, CA, USA). 

### 2.10. Statistical Analysis

One-way or two-way ANOVA were used, respectively, for comparisons of more than two groups with single or multiple time points, followed by a Tukey’s or Dunnett’s post-test to adjust for multiple comparisons. Non-parametric Mann–Whitney test was used for comparisons of two groups. All statistical analyses were performed in GraphPad Prism 8.0. The asterisks shown in the figures refer to the level of significance, * *p* ≤ 0.05; ** *p* ≤ 0.01; *** *p* ≤ 0.001; **** *p* ≤ 0.0001. Serum analysis and viral titer quantification were analyzed in an un-blinded manner.

## 3. Results

### 3.1. Chimeric HA-Based Influenza Vaccination Regimens Induce Durable Stalk-Reactive Immunity in Ferrets

The objective of this preclinical study was to assess the duration of protective immunity induced by the sequential immunization of ferrets with group 1 chimeric hemagglutinins (cHAs) [36,42,43]. Figure 1 illustrates the experimental timeline and immunization regimens for this study. In humans, vaccination with inactivated influenza vaccine induces sub-optimal HA stalk-reactive antibody responses, whereas annual immunization with the influenza vaccine or infection by the influenza virus could induce stalk antibody levels over time [44,45]. To mimic pre-existing HA stalk immunity observed in humans, ferrets were primed by immunization with an influenza B virus expressing cH9/1 (B-cH9/1). Following the prime immunization, we compared the ability of our sequential immunization regimens to boost H1 stalk-specific antibody titers (Figure 1B): (i). Two consecutive LAIV immunizations (LAIV-LAIV regimen), (ii). LAIV followed by inactivated influenza virus vaccine (LAIV-IIV regimen), or (iii). sequential immunization with IIV (IIV-IIV regimen). All IIV vaccines were formulated with AS03, an oil-in-water emulsion-based adjuvant containing tocopherol [46]. To mimic the immunization regimens of our randomized, observer-blinded, phase 1 clinical trial with cHA-based vaccine regimens [47], we included AS03-adjuvanted cH5/1N1 IIV as the booster for the cH8/1N1 LAIV prime immunization. We included cH11/1 LAIV as a second LAIV-based booster immunization for comparisons of vaccine efficacy. Although they are not ideal side-by-side comparisons, the Good Manufacturing Practices (GMP)-grade cH5/1N1 IIV and cH11/1N1 LAIV vaccines are intended for use in clinical trials. One group of ferrets that were immunized with only B-cH9/1 virus was included to assess the level of protective H1 stalk-specific immunity conferred by prime immunization. One group of ferrets immunized twice with seasonal quadrivalent inactivated influenza vaccine (QIV) was included as a “standard of care” control. 

**Figure 2:**
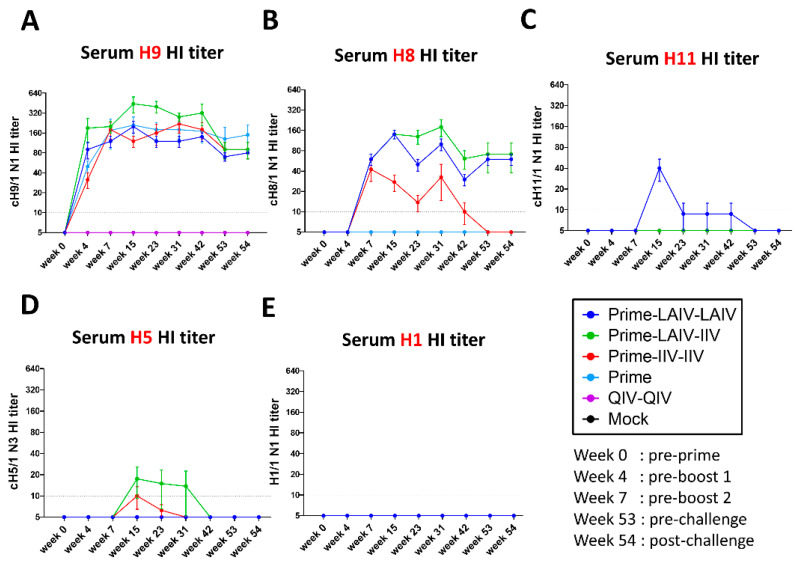
Kinetics of vaccine-specific antibody responses measured by hemagglutination inhibition assay. Hemagglutination inhibition (HI) titers against (**A**) cH9/1, (**B**) cH8/1, (**C**) cH11/1, (**D**) cH5/1 and (**E**) pH1N1 viruses were measured at the indicated time points prior to and following pH1N1 influenza A virus challenge infection. Each y-axis indicates the HI titers against the indicated virus strain. Prime-LAIV-LAIV, Prime-LAIV-IIV, Prime-IIV-IIV, Prime, QIV-QIV, and Mock vaccinated animals are shown as dark blue, green, red, light blue, purple, and black lines, respectively. Mean HI titers ± standard error of the mean (SEM) against the vaccinated virus strains at indicated time points are indicated (*n* = 4 ferrets per group). The black dashed line indicates the limit of detection for the assay.

To assess the kinetics of vaccine-induced antibody responses, serum samples were collected at the indicated time points (Figure 1A), and vaccine-induced antibody responses were evaluated by hemagglutination inhibition assays (Figure 2). All ferrets that received the B-cH9/1 prime immunization developed H9-specific titers ranging from 20 to 640, and HI titers remained at or above 80 for the duration of the study (Figure 2A). All ferrets that received the cH8/1 booster immunizations developed H8-specific titers that ranged from 20 to 160 (Figure 2B). For ferrets that received the LAIV-based cH8/1 vaccine, H8-specific antibody titers were maintained for the duration of the study, whereas H8-specific antibody titers declined to below detectable levels by week 53 for ferrets that received the adjuvanted cH8/1 IIV vaccine. Ferrets that received the cH11/1 LAIV booster immunization developed transient H11-specific antibody titers at week 15, which ranged from 20 to 80 and then declined to below the limit of detection for this assay by week 53 (Figure 2C). Ferrets that received the cH5/1 IIV booster immunization developed low levels of H5-specific antibody titers at weeks 15, 23, and 31, which rapidly declined below the limit of detection for this assay (Figure 2D). During the course of the study, no ferrets developed H1-specific HI titers (Figure 2E). 

**Figure 3:**
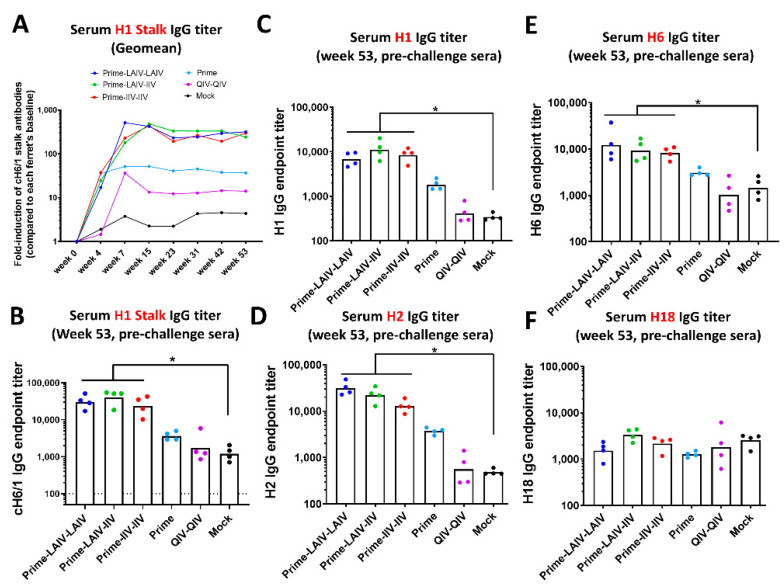
Sequential immunization with cHA-based vaccines induces long-lived broadly reactive HA-specific and H1 stalk antibodies. IgG antibody responses were measured in weeks 0 (pre-prime), 4 (pre-boost1), 7 (pre-boost 2), 15, 23, 31, 42 and/or 53 (pre-challenge). (**A**) Relative to the pre-prime (week 0) serum H1 stalk IgG titers, fold-induction of serum H1 stalk IgG geometric mean endpoint titers against cH6/1 protein at the indicated time points are presented. (**B**) The geometric mean end-point titers of serum H1 stalk IgG on week 53 (pre-challenge) are indicated. The black dashed line indicates the limit of detection for the assay. Serum IgG endpoint titers (y-axis) against (**C**) H1, (**D**) H2, (**E**) H6, and (**F**) H18 were measured prior to pH1N1 challenge infection (week 53). LAIV-LAIV, LAIV-IIV, IIV-IIV, Prime only, and QIV-QIV vaccinated animals are shown as blue, green, red, light blue, and purple, respectively. Mock-immunized animals are shown as black. All animals except for QIV- and mock-immunized ferrets were primed with B-cH9/1 virus. White bars indicate the geometric mean titer (GMT) with individual scatter dot plots. Each point indicates the endpoint titer for each individual ferret (*n* = 4/group). Data in (**B**–**F**) were compared to mock vaccinated animals with non-parametric Mann–Whitney test. The asterisks refer to the level of significance: *: *p* < 0.05 (each of the three cHA-vaccinated groups has similar statistically significant difference as compared to mock group, with *p* = 0.0286).

The kinetics of the H1 stalk-specific and HA-specific antibody responses were measured by enzyme-linked immunosorbent assay (ELISA), which included soluble, recombinant cH6/1, H1, H2, H6, or H18 as capture antigens (Figure 3). LAIV-LAIV, LAIV-IIV and IIV-IIV vaccinated ferrets all had significantly elevated H1 stalk-specific IgG titers. Compared to the baseline of each ferret, all B-cH9/1-primed animals had high induction levels of H1 stalk-specific IgG antibodies, with fold-induction levels ranging from 1:28 to 1:54 at week 4 (pre-boost #1). Importantly, the three groups of ferrets (LAIV-LAIV, LAIV-IIV and IIV-IIV) immunized with cHA boosted anti-stalk antibody titers by week 7 (pre-boost #2). LAIV-LAIV and LAIV-IIV vaccinated ferrets had significantly elevated H1 stalk-specific antibodies as compared to mock-immunized animals on weeks 7, 15, 31, 42, 53 and weeks 15, 42, 53, respectively. No significant differences of H1 stalk-specific antibodies were observed among the LAIV-LAIV, LAIV-IIV, or IIV-IIV groups (Figure 3A). Importantly, all ferrets immunized with cHA-based vaccines developed long-lived H1 stalk antibody responses that were sustained up to week 53 (pre-challenge). During the year-long observation period, ferrets immunized with the Prime-LAIV-LAIV or Prime-LAIV-IIV regimens developed the highest inductions of H1 stalk-specific IgG antibodies, which reached a plateau at week 7 and 15, respectively, and were sustained to week 53 (pre-challenge). In contrast, B-cH9/1 prime-only immunization of ferrets provided a low level of anti-stalk IgG titers. (Figure 3A). A single immunization with QIV, the standard of care, induced low levels of anti-H1 stalk antibodies at week 7 (pre-boost 2), which rapidly declined to levels detected for the mock-immunization controls (Figure 3A). At week 53 (pre-challenge), all cHA-based vaccine regimens induced significantly higher anti-H1 stalk antibodies than the mock immunization regimen (Figure 3B). 

To assess the breadth of antibody responses induced by immunization with the cHA-based vaccines, total IgG antibody titers against homologous H1, and group 1 heterosubtypic H2, H6, and H18 HAs were also measured at week 53 (pre-challenge) by ELISA (Figure 3C–F). All cHA-based vaccination regimens boosted significantly higher H1-specific IgG titers than mock controls; on the contrary, sequential immunization with QIV resulted in H1-specific IgG titers that were comparable to the background levels detected for the mock-immunized control ferrets (Figure 3C). Immunization with the cHA-based vaccine regimens induced significantly higher cross-reactive H2- and H6-specific IgG antibody titers than obtained by mock immunization, following the order of LAIV-LAIV > LAIV-IIV ≥ IIV-IIV (Figure 3D,E). With regards to IgG antibody titers for H18, which is a group 1 HA that is distantly related to H1, there were not significantly different H18-specific IgG antibody titers among all immunization groups (Figure 3F). Taken together, our results indicated that the cHA-based Prime-LAIV-LAIV and/or Prime-LAIV-IIV vaccination approaches boosted antibody responses against the stalk and full-length H1 HA, and boosted broadly cross-reactive IgG antibody responses against group 1 HAs (H1, H2 and H6)-reactive antibodies.

### 3.2. cHA-Based LAIV-LAIV Vaccine Regimen Induced Long-Lived N1 Neuraminidase-Inhibiting Antibodies

**Figure 4:**
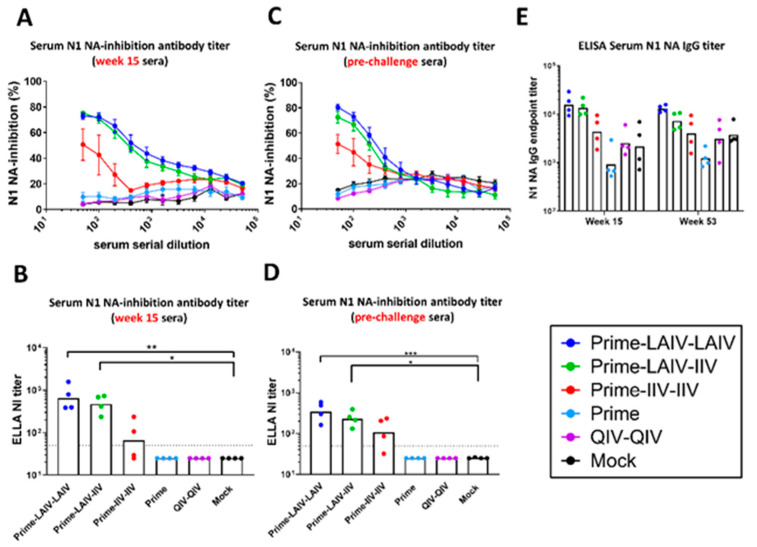
N1 neuraminidase-inhibiting and anti-N1 NA antibody responses measured by ELLA and ELISA. (**A**–**D**) Fetuin-based enzyme-linked lectin assays (ELLA) were used to measure NA-inhibiting (NI)-antibody titers, which are reported as reductions in the N1 NA enzymatic activities of the H7N1 viruses. The N1 NA-inhibition curves on weeks (**A**) 15 and (**C**) 53 (pre-challenge) are graphed. ELLA NI titers were measured at weeks (**B**) 15 and (**D**) 53 (pre-challenge) and are reported as the 50% reduction in the NA enzymatic activities of H7malN1Cal09 virus strain. (**E**) ELISA serum IgG endpoint titers (y-axis) against N1 NA on weeks 15 and 53 (pre-challenge) are indicated. LAIV-LAIV, LAIV-IIV, IIV-IIV, Prime only, and QIV-QIV vaccinated animals are shown as blue, green, red, light blue, and purple, respectively. Mock-immunized animals are shown as black. All animals except for QIV- and mock-immunized ferrets were primed with B-cH9/1 virus. White bars indicate the GMT with individual scatter dot plots. Each point indicates the endpoint titer for each individual ferret (*n* = 4/group). Data were compared to mock vaccinated animals with one-way or two-way ANOVA followed by a Dunnett’s multiple comparison test. The asterisks refer to the level of significance: *: *p* < 0.05; **: *p* < 0.01; ***: *p* < 0.001. The black dashed line indicates the limit of detection for the assay.

Titers of N1 neuraminidase (N1NA)-inhibiting antibodies were measured at the indicated time points, i.e., on weeks 15 and 53 (pre-challenge), by an enzyme-linked lectin assay (ELLA). At week 15, significantly elevated N1 NA-inhibiting antibody titers were observed for the Prime-LAIV-LAIV and Prime-LAIV-IIV vaccination groups as compared to the mock immunization control group, with geometric mean 50% inhibitory concentration (IC50) values of 660 and 471, respectively. The Prime-IIV-IIV immunization regimen also induced weak N1 NA-inhibiting antibody titers (Figure 4A,B). Similar to the pattern observed for the week 15 serum samples, the Prime-LAIV-LAIV immunization regimen, and to a slightly lesser extent the Prime-LAIV-IIV immunization regimen, induced durable neuraminidase-inhibiting antibody titers than did the mock immunized group at week 53 (pre-challenge sera), with geometric mean IC50 values of 345 and 230, respectively. The Prime-IIV-IIV immunized group maintained modest neuraminidase-inhibiting antibody titers with an IC50 value of 88 (Figure 4C,D). Similar patterns of anti-N1 NA IgG titers at weeks 15 and 53 (pre-challenge sera) were observed by ELISA, albeit the mock control group showed a high N1 NA endpoint titer background (Figure 4E). Promisingly, these results imply that the Prime-LAIV-LAIV immunization regimen induces durable neuraminidase-inhibiting antibody titers that are sustained for up to one year. Taken together, our results demonstrated that the Prime-LAIV-LAIV and Prime-LAIV-IIV vaccination approaches induced durable HA stalk-specific, broadly cross-reactive HA IgG and anti-N1 NA antibody responses, which should confer broad protective immunity. 

### 3.3. Vaccination with an LAIV-LAIV Sequential Immunization Regimen Induces Long-Lived Protective Immunity against pH1N1 Viral Challenge

**Figure 5:**
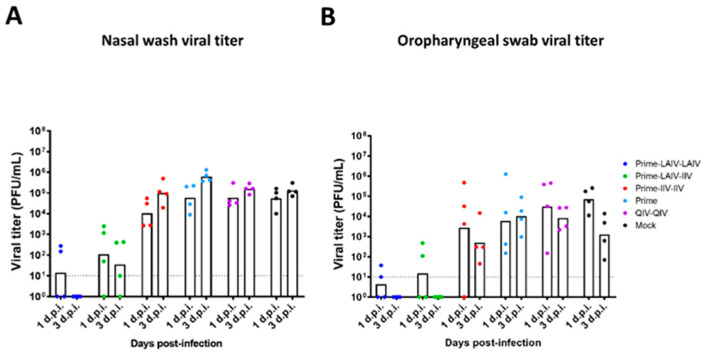
Viral shedding in upper respiratory tract after pH1N1 virus challenge infection. Viral titers for the vaccinated or mock-vaccinated animals were measured by plaque assays. (**A**) Nasal wash and (**B**) Oropharyngeal swab viral titers were measured on days 1 and 3 post-infection with H1N1 influenza A virus. LAIV-LAIV, LAIV-IIV, IIV-IIV, Prime-only, and QIV-QIV vaccinated animals are shown as blue, green, red, light blue, and purple, respectively. Mock-immunized animals are shown as black. All animals except for QIV- and mock-immunized ferrets were primed with B-cH9/1 virus. White bars denote the GMT with individual scatter dot plots. Each point indicates the titer for each individual ferret (*n* = 4/group). The black dashed line indicates the limit of detection for the assay. Data were analyzed by two-way ANOVA followed by a Tukey’s multiple comparison test (multiple time points).

**Figure 6:**
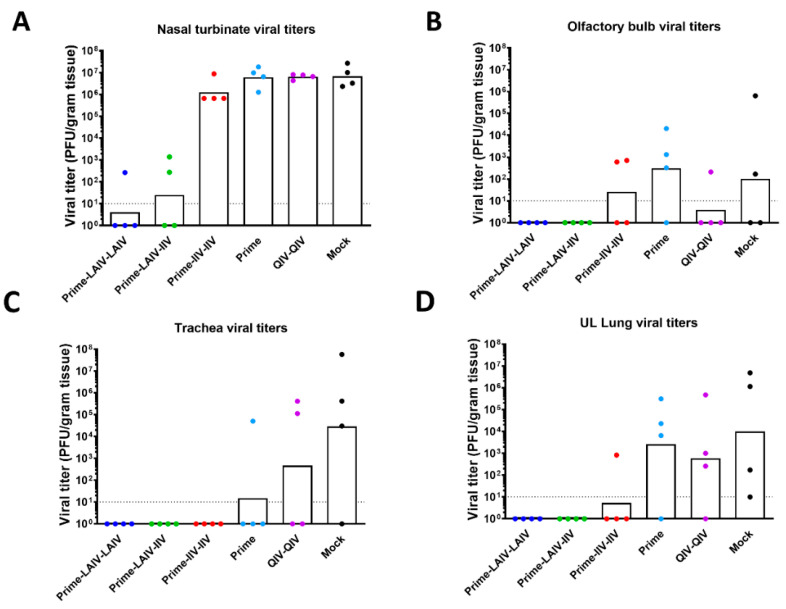
Viral replications following pH1N1 virus challenge infection. Viral titers of vaccinated groups or mock animals were measured by plaque assays. Viral titers from the (**A**) nasal turbinate and (**B**) olfactory bulb of the upper respiratory tract, and viral titers from the (**C**) trachea and (**D**) lung (left medial bronchus of the upper lobe) of the lower respiratory tract were determined on day 4 post-infection. White bars denote the GMT with individual scatter dot plots. Each point indicates the titer for each individual ferret (*n* = 4/group). LAIV-LAIV, LAIV-IIV, IIV-IIV, Prime-only, QIV-QIV vaccinated, and mock animals are shown as blue, green, red, light blue, purple, and black, respectively. The black dashed line indicates the limit of detection for the assay. Data were compared to mock vaccinated animals with one-way ANOVA followed by a Dunnett’s multiple comparison test (single time point).

As illustrated in Figure 1, all ferrets were challenged by intranasal infection with 10^6^ PFU of the pH1N1 influenza A virus, A/California/04/2009 (Cal/09), at 53 weeks post-prime immunization to evaluate the efficacy of protection elicited by the distinct immunization regimens. On days 1 and 3 post-challenge infection, nasal wash and oropharyngeal swab samples were collected to examine the kinetics of virus replication in the upper respiratory tract (Figure 5). On day 4 post-challenge infection, all animals were humanely euthanized for the collection of respiratory tract specimens for subsequent analysis of viral titers (Figure 1 and Figure 6). As expected from our previous pre-clinical study [29], the Prime-LAIV-LAIV sequential vaccination regimen provided protective immunity to reduce viral replications in the upper respiratory tract. Ferrets immunized with the Prime-LAIV-LAIV vaccination regimen developed low influenza virus titers (GMT of 1.4 × 10^1^ PFU/mL), which could only be detected at day 1 post-infection and were below the limit of detection at day 3 post-infection (Figure 5A). Ferrets that were sequentially immunized with the LAIV and AS03-adjuvanted IIV vaccine experienced reduced levels of virus replication in the upper respiratory tract. In ferrets immunized with the Prime-IIV-IIV vaccine regimen, we observed no obvious decrease in viral replication within the upper respiratory tract on either day post-challenge. High levels of virus replication were observed on both sampling days for the mock-immunization, B-cH9/1 virus (prime only), and QIV-QIV immunization groups (Figure 5A). Similar trends in virus titers were also observed for the oropharyngeal swab samples, albeit the viral titers were lower than for the nasal wash specimens (Figure 5B).

We also evaluated viral replication within tissues from the upper and lower respiratory tracts on day 4 post-challenge infection with pH1N1 influenza virus. Encouragingly, the Prime-LAIV-LAIV and, to a slightly lesser extent, Prime-LAIV-IIV vaccinated ferrets effectively suppressed virus replication within nasal turbinates, with GMTs of 4 and 24.8 PFU/gram tissue, respectively. However, high levels of virus replication were detected from the Prime-IIV-IIV, B-cH9/1 virus (prime only), QIV-QIV, and mock-immunized groups, with GMTs of 1.26 × 10^6^, 6.20 × 10^6^, 6.50 × 10^6^, and 6.75 × 10^6^ PFU/gram tissue, respectively (Figure 6A). Whereas olfactory bulb virus titers were below the limit of detection for the Prime-LAIV-LAIV and Prime-LAIV-IIV immunization groups, low levels of virus replication within this tissue could be detected in some ferrets from the Prime-IIV-IIV, Prime, QIV-QIV, and mock-immunization groups (Figure 6B). Except for one Prime-IIV-IIV-vaccinated ferret with a titer of 8.2 × 10^2^ PFU/gram lung tissue, all cHA-based vaccines induced sterilizing immunity that protected ferrets against viral replication within the trachea and lung (Figure 6C,D). Immunization of ferrets with the B-cH9/1 prime-only and QIV-QIV vaccination regimens considerably reduced viral replication in the trachea as compared to mock controls, with GMTs of 1.5 × 10^1^, 4.64 × 10^2^, and 2.92 × 10^4^ PFU/gram tissue, respectively (Figure 6C). However, viral titers from upper left (UL) lung specimens were not significantly decreased for the B-cH9/1 prime-only and QIV-QIV immunization groups as compared to mock-immunized animals, with GMTs of 2.6 × 10^3^, 5.89 × 10^2^, and 9.84 × 10^3^ PFU/gram tissue, respectively (Figure 6D). Taken together, our results demonstrated that, as compared to the other immunization regimens tested in this study, the Prime-LAIV-LAIV vaccination regimen induced superior HA stalk-specific and anti-N1 NA antibody responses, which conferred the best protection against pH1N1 virus infection.

### 3.4. cHA-Based Vaccination Regimens Stimulated Activation of T Cell Immune Responses in Ferrets

**Figure 7:**
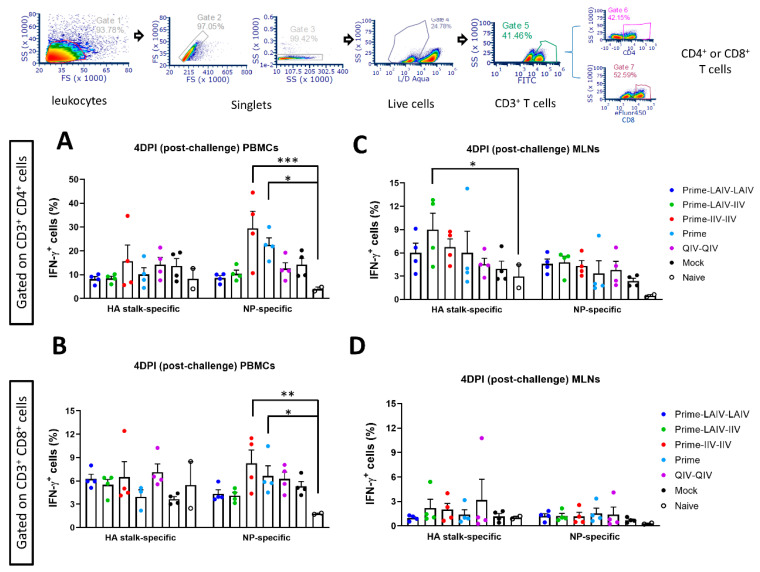
Flow cytometric gating scheme and analysis of influenza-specific T cell responses in peripheral blood mononuclear cells (PBMCs) and mediastinal lymph nodes (MLNs). The gating strategies for immunophenotyping of immune cells by a Gallios flow cytometer are indicated in the top panel. Singlets of lymphocytes (gated by SSC-A vs. FSC-A, followed by FSC-H vs. FSC-W and SSC-W vs. SSC-H) and live cells (based on Live/Dead Aqua stain) were acquired for analysis of T cell responses. CD3+, CD4+, and CD8+ T cells were isolated by distinct fluorescent dyes. Gated on the CD3+CD4+ cells, percentages of pH1N1 HA stalk-specific or NP-specific IFN-γ^+^ cells in PBMCs (**A**) and MLN (**C**) were plotted, respectively. Gated on the CD3+CD8+ cells, percentages of pH1N1 HA stalk-specific or NP-specific IFN-γ^+^ cells in PBMCs (**B**) and MLN (**D**) were plotted, respectively. LAIV-LAIV, LAIV-IIV, IIV-IIV, Prime only, and QIV-QIV vaccinated animals are shown as blue, green, red, light blue, and purple, respectively. Mock-immunized animals are shown as black. All animals except for QIV and mock groups were primed with B-cH9/1 virus. Each point indicates the percentage of IFN-γ^+^ cells for each individual ferret (*n* = 4/group). The asterisks refer to the level of significance: *: *p* < 0.05; **: *p* < 0.01; ***: *p* < 0.001.

Since the contributions of influenza virus-specific cellular immune responses are crucial for cross-protective and long-lasting protective immunity [48], herein, besides humoral immunity above, we also examined the effector T cell responses of peripheral blood mononuclear cells (PBMCs) and mediastinal lymph nodes (MLN) obtained from the ferrets who received distinct immunization regimens. On day 4 post-pH1N1 virus challenge infection, significantly higher percentages of nucleoprotein (NP)-specific effector T cells (both CD3^+^CD4^+^IFN-γ^+^ and CD3^+^CD8^+^IFN-γ^+^) were observed in Prime-IIV-IIV and B-cH9/1 virus (Prime-only) immunized PBMCs as compared to those from naïve ferrets (Figure 7A,B). Alternatively, Prime-LAIV-IIV immunization induced more robust HA stalk-specific CD4+ effector T cells (CD3^+^CD4^+^IFN-γ^+^) in MLNs than that of naïve ferrets (Figure 7C). All cHA-based and QIV-QIV vaccine groups also induced slightly higher percentages of NP-specific effector T cells (both CD3^+^CD4^+^IFN-γ^+^ and CD3^+^CD8^+^IFN-γ^+^) in MLNs as compared to the naïve group (Figure 7C,D). Our results showed that NP-specific IFN-γ^+^ effector T cell responses (i.e., the response against a replicating virus) in PBMCs (both CD4 and CD8) were highest in the four groups where the challenge virus replicated to significant levels (as shown in Figure 6A), while the highest HA-specific responses in MLNs were observed among CD4 cells from the three cHA-vaccinated groups (the highest being observed in the LAIV-IIV regimen) (Figure 7). These results suggest a possible correlation between the two NP-specific responses (CD4 and CD8) and the level of virus replication. These findings suggested that distinct cHA-based vaccination approaches resulted in differential cellular immune regulatory mechanisms, i.e., localized (draining lymph node) or systemic immunity, to correlate to the protection against pH1N1 virus infection.

## 4. Discussion

In recognition of the poor vaccine efficacy of seasonal influenza vaccines and the public health threat of influenza viruses with pandemic potential, the National Institute of Allergy and Infectious Diseases (NIAID) of the U.S. National Institutes of Health (NIH) released a directive to develop universal influenza vaccines (UIV) [20]. The ideal UIV should have improved effectiveness in protecting against symptomatic influenza virus infection, protect against seasonal and pandemic influenza viruses, and confer durable protective humoral immunity that lasts at least one year [21,22,23]. We have developed a chimeric hemagglutinin (cHA)-based universal influenza virus vaccine that is designed to focus humoral immunity on the highly conserved HA stalk domain [28,29]. We previously reported on the efficacy of our cHA-based UIV to protect against infection of group 1 and group 2 influenza A viruses and influenza B viruses in the mouse and/or ferret models of influenza. In this study, we assessed in the ferret model the duration of H1 HA stalk-specific antibody responses induced by sequential immunization with our cHA-based vaccines and examined the breadth of protective immunity induced by our sequential immunization regimens. All ferrets that were prime immunized with the B-cH9/1 vaccine developed vaccine-specific HI titers that persisted for the duration of the study (Figure 2). Ferrets that received cH8/1 LAIV developed long-lived H8-specific antibody titers. However, ferrets that were immunized with adjuvanted cH8/1 IIV or cH5/1 IIV developed short-lived H8- or H5-specific HI antibody titers, respectively. Consistent with our previous findings [31,33], the Prime-LAIV-LAIV, Prime-LAIV-IIV, and Prime-IIV-IIV vaccination regimens induced comparable broadly reactive and H1 stalk-specific humoral immune responses (Figure 3). Furthermore, the Prime-LAIV-LAIV and Prime-LAIV-IIV vaccination regimens induced durable neuraminidase inhibiting (NAI) antibody titers that were moderately higher than those induced by the Prime-IIV-IIV vaccine regimen (Figure 4). Although the overall trends matched our expectations for cross-reactive anti-HA and anti-N1 NA IgG antibody responses, the antibody titers detected by ELISA were uniquely elevated for the mock group, which may result from variation in antibody binding for different batches of recombinant proteins (Figure 3 and Figure 4E). These results suggest that the sequential immunization of ferrets with our cHA-based universal influenza vaccine induced H1 stalk-specific and N1-specific antibody responses that persisted for up to one-year post immunization, which meets one of the criteria for a universal influenza vaccine [20]. 

At 53 weeks post-prime immunization, all ferrets were challenged by intranasal infection with a pH1N1 influenza A virus. In agreement with our previous findings [31,33], the Prime-LAIV-LAIV immunization regimen was superior to the other immunization regimens in conferring protection against infection by the pH1N1 influenza virus. Low virus titers could be detected in nasal wash and oropharyngeal swab specimens only on day 1 post-infection of the ferrets that received the Prime-LAIV-LAIV immunization regimen. Additionally, consistent with our previous findings [31,33], reduced virus titers were detected in both upper and lower respiratory tract specimens from ferrets that received the Prime-LAIV-IIV immunization regimen. The high viral titers detected in the respiratory tract specimens obtained from the Prime-IIV-IIV, Prime, and QIV-QIV immunized ferrets suggested that at 46 weeks following the last vaccination, these immunization regimens did not confer protection against infection by the pH1N1 influenza virus (Figure 5). Consistently, the Prime-IIV-IIV immunization regimen conferred little protection against the pH1N1 infection, despite the induction of H1 stalk-specific serum antibody responses [33]. These results support our previous conclusions that mucosal antibody responses in concert with cellular immunity are required for complete protection against infection by influenza A virus [31]. As for T cell responses, our results showed that the cHA-based IIV-IIV, but not LAIV-LAIV or LAIV-IIV, regimen recalled potent NP-specific effector T cells in PBMCs on day 4 post-pH1N1 challenge infection (Figure 7). The lower amounts of NP-specific effector T cells that were observed in PBMCs of LAIV-LAIV or LAIV-IIV vaccinated ferrets may result from the virus being completely cleared or significantly suppressed during the early stage of infection (days 1 to 3 post-challenge infection) (Figure 5), and thus no further T cell expansion was required to fight the infection. Similar findings were also demonstrated in the mouse model [49]. Alternatively, cHA-based vaccine approaches, especially for the LAIV-IIV regimen, recalled more robust HA stalk- and/or NP-specific CD4+ effector T cells (CD3+CD4+IFN-γ^+^) in lung draining lymph node, i.e., MLNs, but not in circulating PBMCs, than that of naïve ferrets (Figure 7), which may correlate to better protection in the lung (Figure 6). Similarly, Yang and colleagues demonstrated that the combined use of inactivated and live-attenuated vaccines induced robust HA-specific memory recall responses from bronchoalveolar lavage after heterologous virus challenge in the mouse model [50]. In addition to effector T cells, we hypothesized that recall of memory B cell or T cell subsets, such as tissue-resident memory T cells, long-lived plasma cells, or memory B cells, contributes to the persistent protective immunity against pH1N1 virus challenge infection. The immunological basis of these protective mechanisms in ferrets requires further investigation. With respect to virus replication within the respiratory tract, the Prime-LAIV-LAIV and Prime-LAIV-IIV immunization regimens conferred comparable protective immunity against influenza virus replication within respiratory tract tissues (Figure 6). Consistent with the nasal wash virus titers and our previous findings [33], the Prime-IIV-IIV immunization regimen did not prevent virus replication within the nasal cavity. However, this immunization regimen was more effective in reducing virus replication within the lower respiratory tract than we previously observed [33]. These discrepancies could be attributed to the potential induction of higher NAI antibody titers within the respiratory tract in the current study.

As mentioned previously, the U.S. NIAID outlined three essential criteria for a universal influenza vaccine [20]. We previously reported on the efficacy of our sequential immunization regimen based on cHA-based LAIV vaccines to provide immunity against infection with human and avian influenza A viruses [31,32,33,34], which addresses two of the three UIV criteria established by the NIAID. Herein, we demonstrated that a sequential immunization regimen of cHA-based LAIV vaccines satisfied the third criterion for a UIV by inducing long-lived humoral and cellular immune responses, which provided protection against challenge infection by a pH1N1 influenza A virus. Our current findings are consistent with our previous reports [31,33], and are in agreement with those obtained by Rudenko and colleagues from the mouse and ferret models of influenza [35,51]. The results obtained in this study with naïve ferrets suggest that of the three sequential immunization regimens examined, the Prime-LAIV-LAIV immunization regimen is the most promising regimen for conferring protection against influenza A viruses encoding group 1 type hemagglutinin. Collectively, our findings demonstrate that our cHA-based influenza virus vaccines, which are designed to refocus immune responses on the conserved HA stalk domain, are a viable approach to a universal influenza virus vaccine. Our cHA-based UIV progressed to first-in-human Phase I studies. The interim analyses of this randomized, observer-blinded, phase 1 study of group 1 cHA vaccines showed that LAIV followed by AS03-adjuvanted IIV vaccination induced stalk-specific antibodies [47]. Our findings from our preclinical ferret studies echo the results described in the final report of that placebo-controlled phase I trial (NCT03300050), which indicated that sequential immunization with our cHA-based vaccine approaches elicited cross-reactive and durable anti-HA stalk antibodies over a year in healthy 18~39-year-old adult vaccine recipients [52]. Although our results from the ferret model of influenza are encouraging, there are some caveats to consider. Naïve ferrets do not model the complex influenza immune landscape of humans, especially for the elderly populations. Pre-existing influenza immunity in humans could explain the strong H18 antibody responses, which were less pronounced in the naïve ferrets. In addition, it is plausible that the germline B cell repertoire of ferrets is genetically limited, which could result in a narrower humoral response against the conserved hemagglutinin stalk domain [53].

## 5. Conclusions

In this study, we compared the efficacy and duration of protective immunity elicited by our cHA-based vaccine regimens. We concluded that sequential immunizations with cHA-based LAIV-LAIV and LAIV-IIV immunization regimens induced long-lived stalk-specific and broadly reactive humoral and cellular immune responses. The LAIV-LAIV immunization regimen conferred the best protection against a pH1N1 virus challenge infection in ferrets. 

## Data Availability

The data presented in this study are available on request from the corresponding author.

## Institutional Animal Care and Use Committee Statement

All animal experiments in this study were performed in accordance with an animal protocol (IACUC-2013-1408) approved by the Institution Animal Care and Use Committee (IACUC) of the Icahn School of Medicine at Mount Sinai (ISMMS).
